# Early recognition of sepsis: an audit of sepsis diagnosis and initial management

**DOI:** 10.1186/2197-425X-3-S1-A347

**Published:** 2015-10-01

**Authors:** JB Simon, R Azim, P Vondras

**Affiliations:** Conquest Hospital, Intensive Care Department, Hastings, United Kingdom

## Introduction

Early recognition and management of sepsis is associated with improved mortality and reduced critical care admissions [1]. International campaigns have tried to improve sepsis outcomes, most notably the surviving sepsis campaign which has a group of interventions i.e. the “3 hour and 6 hour bundles” at its core [2]. By improving adherence to these, mortality and morbidity associated with sepsis can be improved.

The VitalPAC interface, introduced to our hospital in 2014, is an electronic system used as a replacement to conventional patient records, which allows remote viewing of patient data.

## Objectives

We aimed to analyse the speed of sepsis diagnosis, and the rate at which the interventional bundles were being delivered to patients across the hospital site, in an inpatient setting.

## Methods

We evaluated patients meeting the diagnostic criteria for sepsis across all in-hospital specialities over a month, using the VitalPAC system to randomly identify patients. Once selected, the time at which they initially met the criteria was compared to when sepsis was first diagnosed, and the time taken to complete the interventions in the initial 3-hour bundle was assessed.

## Results

Diagnosis and management of 72 patients was evaluated, and a wide variation in management observed. Only 19% had the 3 hour bundle completed on time, and 58% had 2 or fewer parts complete at 3 hours after becoming septic. Measurement of serum lactate and obtaining blood cultures were the worst completed aspects (36% and 42% respectively).

Speed in recognition of the septic patient was also variable - from diagnosis being made before SIRS criteria had been met, to 56h after. In 16 cases no formal diagnosis of sepsis was made, and this group had the highest mortality rate - 53% compared to 28% overall.

When number of components of the 3 hour bundle was analysed, it revealed that those with all 4 interventions complete had the highest mortality rate - 36%, compared to an overall mortality of 28%. However they had the lowest rate of critical care admissions - 0% compared to 13% overall.

## Conclusions

Both recognition of sepsis and adherence to the 3 hour bundle of sepsis interventions within the audit population was poor. If improvements in these aspects are made, the rate of critical care admissions and mortality could be reduced. We intend to implement changes including greater awareness, improved use of the VitalPAC observation software, and a sepsis proforma to guide the early management. Once implemented, we intend to re-audit to determine if improvements in outcomes have been made.
